# Anti-Inflammatory Effects Exerted by 14-Methoxyalternate C from Antarctic Fungal Strain *Pleosporales* sp. SF-7343 via the Regulation of NF-κB and JAK2/STAT3 in HaCaT Human Keratinocytes

**DOI:** 10.3390/ijms232314642

**Published:** 2022-11-24

**Authors:** Linsha Dong, Thao Quyen Cao, Zhiming Liu, Nguyen Quoc Tuan, Youn-Chul Kim, Jae Hak Sohn, Joung Han Yim, Dong-Sung Lee, Hyuncheol Oh

**Affiliations:** 1 College of Pharmacy, Chosun University, Dong-gu, Gwangju 61452, Republic of Korea; 2 Institute of Pharmaceutical Research and Development, College of Pharmacy, Wonkwang University, Iksan 54538, Republic of Korea; 3 Hanbang Cardio-Renal Syndrome Research Center, Wonkwang University, Iksan 54538, Republic of Korea; 4 College of Medical and Life Sciences, Silla University, Busan 46958, Republic of Korea; 5 Division of Polar Life Sciences, Korea Polar Research Institute, Incheon 21990, Republic of Korea

**Keywords:** Antarctic fungi, skin inflammation, JAK2/STAT3, NF-κB, HaCaT

## Abstract

Atopic dermatitis (AD) is a chronic inflammatory skin disease with a profound negative impact on patients’ quality of life. Four known secondary fungal metabolites were found in the chemical study of the Antarctic fungus *Pleosporales* sp. SF-7343, including 14-methoxyalternate C (**1**), 5′-methoxy-6-methyl-biphenyl-3,4,3′-triol (**2**), 3,8,10-trihydroxy-4-methoxy-6-methylbenzocoumarin (**3**), and alternariol monomethyl ether (**4**). Additionally, we identified the skin anti-inflammatory composition from the SF-7343 strain. Interleukin-8 and -6 Screening results showed that compound **1** inhibited IL-8 and IL-6 in tumor necrosis factor-α/interferon-γ stimulated HaCaT cells. Compound **1** showed inhibitory effects on MDC and RANTES. It also downregulated the expression of intercellular adhesion molecule-1 (ICAM-1) and upregulated the expression of involucrin. The results of the mechanistic study showed that compound **1** inhibited the nuclear translocation of nuclear factor-kappa B p65 and STAT3. In conclusion, this study demonstrates the potential of the Antarctic fungal strain SF-7343 as a bioactive resource to inhibit skin inflammation, such as AD.

## 1. Introduction

Atopic dermatitis (AD) is a usual chronic skin inflammatory disease. Approximately 10% of adults and 20% of children suffer from this disease, which is characterized by a compromised immune system, excessive inflammation, and skin barrier disruption [1,2]. Genetic factors, immune disorders, and epidermal barrier dysfunction are all causes of AD, the pathogenesis is very complex. Severe AD is now generally treated with monoclonal antibodies. Long-term use of steroids and antihistamines has significant side effects. Skin fragility and thinning, suppression of melanocytes and gastrointestinal side effects. Therefore, there is a need to find alternative strategies for the treatment of mild or moderate AD [3,4,5]. In many instances, skin diseases caused by infections or other tissue damage are indistinguishable from inflammation [6]. The two major causes of AD are skin immune system disorder and epidermal barrier destruction [7]. A damaged epidermal barrier allows the intrusion of various allergens, triggering immune imbalance and aggravating the development and deterioration of AD. At the onset of AD, resident and infiltrating cells, such as keratinocytes, langerhans cells, and neutrophils, overexpress chemokines [8,9], which activate type 2 helper T (Th2) cells and induce the generation of Th2-type cytokines. This results in the aggravation of the epidermal barrier, impaired keratinocyte differentiation, persistent skin itching, and impairment of stratum corneum permeability [10]. Keratinocytes are the dominant kind of type in the epidermis cells. Compounds with anti-inflammatory effects play a key role in the progress and pathogenesis of AD by enabling communication with other cells. Hence, *in vitro* keratinocyte models have been widely used to study the possibility of various natural and synthetic substances as anti-inflammatory candidates to regulate inflammation in the keratinocytes [11].

The Antarctic microorganisms are of particular interest because of the enormous potential for isolating new biologically active and valuable components that are yet unexplored [12]. This can be attributed to the unique and harsh environment of the Antarctic, which induces unusual metabolic properties and the production of unusual metabolites [13]. In our previous studies, we isolated four compounds from the metabolites of *Pleosporales* sp. SF-7343, and elucidated the anti-inflammatory effects of alternate C in skin inflammation in human keratinocytes [14]. In this study, we isolated additional components from the fungal strain *Pleosporales* sp. SF-7343 and investigated the regulation of skin inflammatory response in human keratinocytes exerted by these compounds.

## 2. Results

### 2.1. Determination of the Molecular Structure of the Isolated Compounds from Metabolites

**Figure 1:**
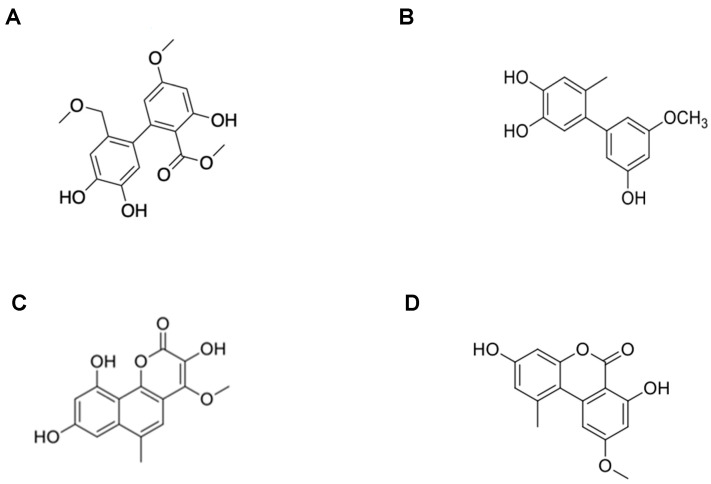
Structure of isolated compounds **1**–**4** (**A**–**D**).

To obtain 14-methoxyalternate C (**1**), 5′-methoxy-6-methyl-biphenyl-3,4,3′-triol (**2**), 3,8,10-trihydroxy-4-methoxy-6-methylbenzocoumarin (**3**), and altenuene (**4**). Multi step chromatographic analysis were carried on using the fungal strain dry fermentation extract. Based on comparing the results of 1D and 2D NMR and MS analyses with data reported in the literature, their structures are shown in Figure 1.

Compound **1**, based on the HRESIMS peak at *m/z* 357.0956 [M + Na]^+^ (calcd. for C_17_H_18_O_7_Na, 357.0950) the molecular formula was determined to be C_17_H_18_O_7_. The ^13^C-, DEPT-, and HMQC- NMR spectra of compound **1** revealed signals of a carbonyl carbon (δ_C_ 171.5), 12 olefinic carbons [containing four oxygenated carbons at δ_C_ 163.6, 162.9, 145.1, and 144.6], three methoxy carbons (δ_C_ 57.5, 55.8, and 52.0), and an oxygenated methylene carbon (δ_C_ 72.5). The requied 9 degrees unsaturation, these molecular formulas occupied for 7, thereby indicating that compound **1** is a biphenyl derivative. Additionally, the NMR data of compound **1** were quite similar to those of alternate C [15], suggesting that compound **1** had the same biphenyl skeleton made of two tetrasubstituted benzenes. A detailed comparison of the 1D NMR data of compound **1** and alternate C revealed that the difference in the signal is due to the presence of methoxymethylene protons instead of a methyl group at C-13. This structural difference was supported by the Heteronuclear Multiple Bond Correlation (HMBC) of H-12/C-14, H-14/C-13, C-14-OCH_3_, and H-14-OCH_3_/C-14 (Table S1). Therefore, the structure of Compound **1** is thought to be 14-methoxyalternate C.

Compound **2**, the molecular formula C_14_H_14_O_4_ was established based on the peak observed at *m/z* 245.0817 [M–H]^−^ (calcd. for C_14_H_13_O_4_, 245.0814) in the HR-ESI-MS spectrum. The ^1^H NMR spectrum of compound **2** displayed five olefinic methine protons at δ_H_ 6.70 (1H, s, H-5), 6.68 (1H, s, H-2), 6.35 (2H, overlapped, H-2′, H-4′), and 6.31 (1H, d, J = 2.0 Hz, H-6′), a methoxy group at δ_H_ 3.76 (3H, s, OCH_3_-3′), and a methyl group at δ_H_ 2.11 (3H, s, CH_3_-6). The ^13^C and DEPT NMR spectra revealed 12 olefinic carbon signals [containing four oxygenated quaternary carbons at δ_C_ 161.6 (C-5′), 159.0 (C-3′), 145.1 (C-3), and 143.6 (C-4); three quaternary carbons at δ_C_ 145.1 (C-1′), 134.4 (C-1), and 126.9 (C-6); and five methine carbons at δ_C_ 118.0 (C-5), 117.4 (C-2), 109.8 (C-2′), 107.2 (C-6′), and 100.3 (C-4′)], a methoxy carbon at δ_C_ 55.5 (3H, s, OCH_3_-3′), and a methyl carbon at δ_C_ 19.8 (3H, s, CH_3_-6) (Table S2). A comparison of the 1D NMR data of compound **2** in the literature helped determine the structure of compound **2**, as shown in Figure 1B [16].

Compound **3** was determined to be C_15_H_12_O_6_ by observing the peak at *m/z* 287.0545 [M–H]^−^ (calcd. for C_15_H_11_O_6_, 287.0550) in the HR-ESI-MS spectrum. The ^1^H NMR spectrum of compound **3** displayed three olefinic methine protons at δ_H_ 7.27 (1H, d, J = 2.2 Hz, H-7), 6.79 (1H, s, H-5), and 6.54 (1H, d, J = 2.2 Hz, H-9), a methoxy group at δ_H_ 3.94 (3H, s, OCH_3_-4), and a methyl group at δH 2.69 (3H, s, CH_3_-6) (Table S3). Compound **3** was identified as 3,8,10-trihydroxy-4-methoxy-6-methylbenzocoumarin. [17]

The molecular formula of Compound **4** was established based on the peak observed at *m*/*z* 295.0580 [M + Na]^+^ (calcd. for C_15_H_12_O_5_Na; 295.0577) in the HR-ESI-MS spectrum. The ^1^H NMR spectrum of compound **4** displayed four olefinic methine protons at δ_H_ 7.22 (1H, d, J = 2.0 Hz, H-6), 6.72 (1H, d, J = 2.4 Hz, H-5′), 6.64 (1H, d, J = 2.4 Hz, H-3′), and 6.61 (1H, d, J = 2.0 Hz, H-4), and a methyl group at δ_H_ 2.73 (3H, s, CH3-6′). The ^13^C and DEPT NMR spectra revealed 15 carbons containing a carbonyl carbon at δC 166.2 (C-7), 12 olefinic carbons [including four methine carbons at δC 117.7 (C-5′), 103.3 (C-6), 101.6 (C-3′), and 99.1 (C-4)], a methoxy carbon at δC 55.8 (OCH_3_-5), and a methyl carbon at δC 25.0 (CH_3_-6′) (Table S4). Compound **4** was identified as an alternariol monomethyl ether [18].

### 2.2. Cell Viability of Isolated Compounds ***1***, ***2***, ***3***, and ***4*** in HaCaT Cells

**Figure 2:**
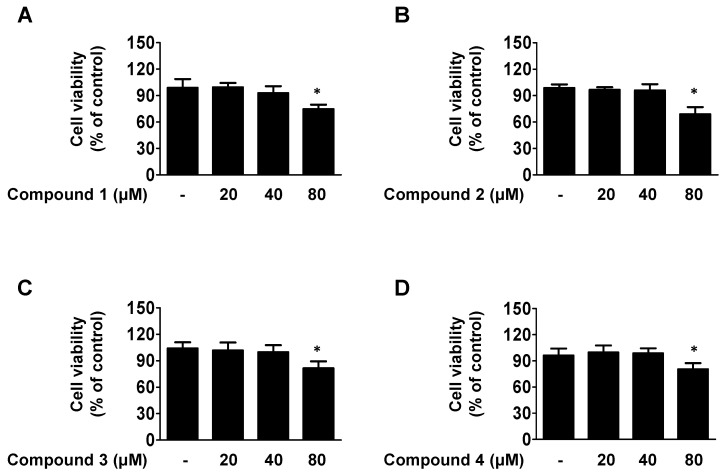
Cytotoxicities of the compounds **1**, **2**, **3**, and **4** used in this study(**A**–**D**). Cells were treated for 24 h with the indicated concentration of each of the four compounds and the cytotoxicity was evaluated. Data are presented as the mean ± SD (n = 3). * *p* < 0.05 vs. control.

We using a MTT assay to examined the cytotoxicity of our isolated compounds **1**, **2**, **3**, and **4**. Results are shown in Figure 2; for the subsequent experiments, the cells were co-treated with these four compounds in a safe concentration (10–40 μM). 

### 2.3. Inhibitory Effects of the Four Compounds on IL-6 and IL-8 Production in TNF-α/IFN-γ-Treated HaCaT Cells

The secretion of IL-6 and IL-8 were to select the compound which we isolated is with anti-inflammatory effect. The results are shown in Table 1, wherein compound **1** inhibited the secretion of IL-8 and IL-6. However, compounds **2**, **3**, and **4** had no obvious inhibitory effect on IL-8 or IL-6 secretion. Therefore, we performed further experiments using only compound **1**. 

### 2.4. Effects of Compound ***1*** on the Level of MDC and RANTES in TNF-α/IFN-γ-Stimulated HaCaT Cells

**Figure 3:**
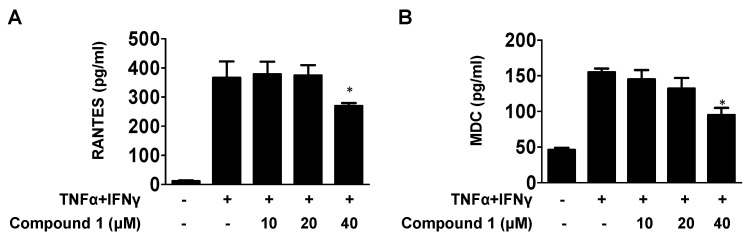
Effects of compound **1** on MDC and RANTES secretion in TNF-α/IFN-γ-stimulated HaCaT cells. (**A**,**B**) The MDC and RANTES levels were measured using the cell cuture supernatant. Data are represented as the mean ± SD (n = 3). * *p* < 0.05 vs. TNF-α/IFN-γ-treated group.

The main fuction of chemokines is to regulate the recruitment of inflammatory cells at the site of infection or inflammation. The effects of compound **1** on RANTES and MDC are shown in Figure 3A,B, wherein the secretion of MDC and RANTES was significantly increased by co-stimulated TNF-α and IFN-γ compared to that in the control groups, which showed a dose-dependent reduction on pretreatment with compound **1**.

### 2.5. Effects of Compound ***1*** on TNF-α/IFN-γ-Induced ICAM-1 and on Barrier-Related Molecules, FLG/IVL in HaCaT Cells

**Figure 4:**
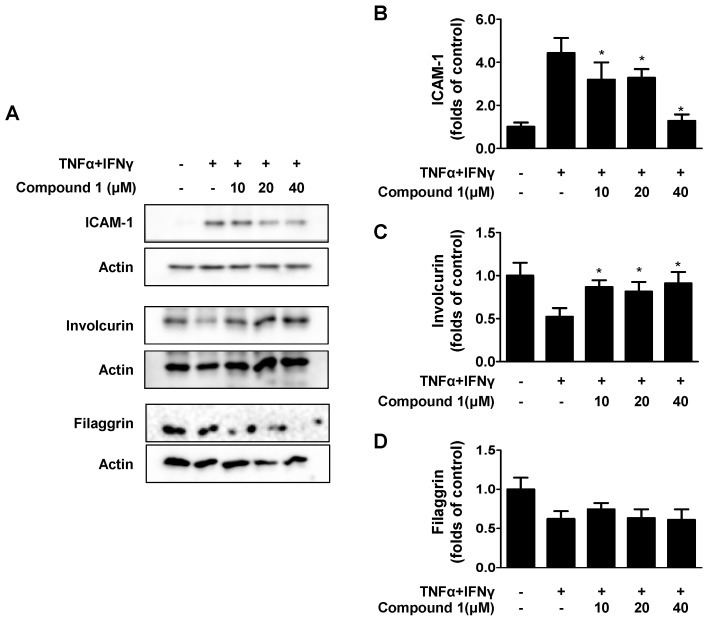
Effects of compound **1** on TNF-α/IFN-γ-induced ICAM-1 and barrier-related molecules, Filaggrin (FLG)/Involucrin (IVL) in HaCaT cells (**A**–**D**). Data are represented as the mean ± SD (n = 3). * *p* < 0.05 vs. TNF-α/IFN-γ-treated group.

ICAM-1 is an important kind of lesion-recruiting adhesion molecule. In AD skin lesions, the overexpression of ICAM-1 can be observed [19]. The effects of compound 1 on TNF-α- and IFN-γ-induced ICAM-1 expression were examined. As shown in Figure 4A,B, compound **1** downregulated ICAM-1 expression. Filaggrin (FLG) and involucrin (IVL) are essential for maintaining epidermal barrier function [20]. As shown in Figure 4A,C,D, compound **1** did not affect FLG expression in TNF-α- and IFN-γ-induced HaCaT cells. In contrast, compound **1** upregulated IVL expression.

### 2.6. Effects of Compound ***1*** on the JAK2/STAT3 Signaling Pathways in HaCaT Cells

**Figure 5:**
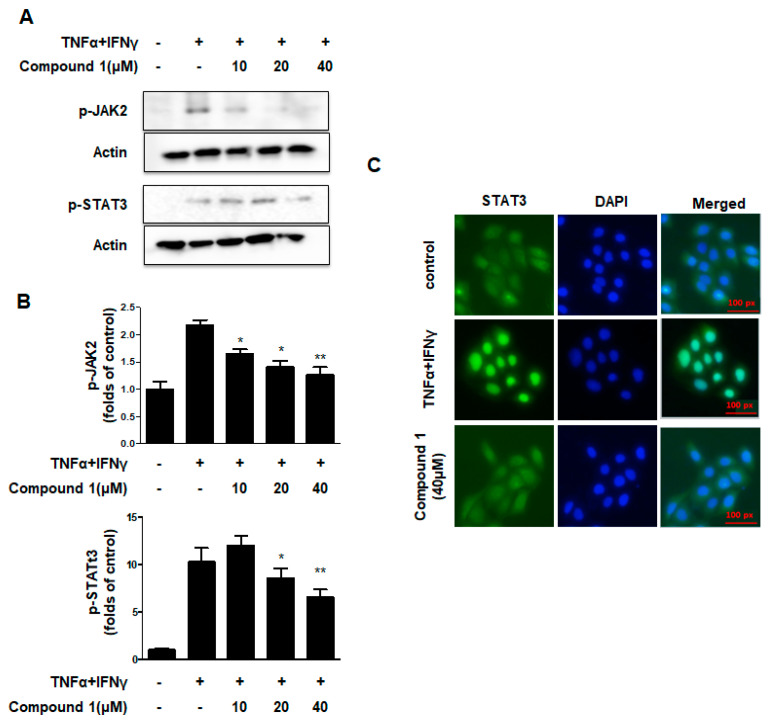
Effects of compound **1** on the JAK2/STAT3 signaling pathways in HaCaT cells. (**A**–**C**) The expression of p-STAT3 and p-JAK2 were measured using Western blotting. Data are represented as the mean ± SD (n = 3). * *p* < 0.05, ** *p* < 0.01 vs. TNF-α/IFN-γ-treated group.

JAK/STAT pathway regulates the major signaling cascades for many cytokines, chemokines and growth factors, and it also participates in intracellular signal transduction and expression [21]. As shown in Figure 5A,B, when cells were co-treated with TNF-α and IFN-γ, compound **1** significantly downregulated the phosphorylation of JAK2 and STAT3. As shown in Figure 5C, the nuclear translocation of STAT3 was inhibited by compound **1**, consistent with the results of Western blotting. These results suggest that compound **1** regulates the JAK2/STAT3 signal pathways in HaCaT cells.

### 2.7. Effects of Compound ***1*** on NF-κB Signaling Pathways in HaCaT Cells

**Figure 6:**
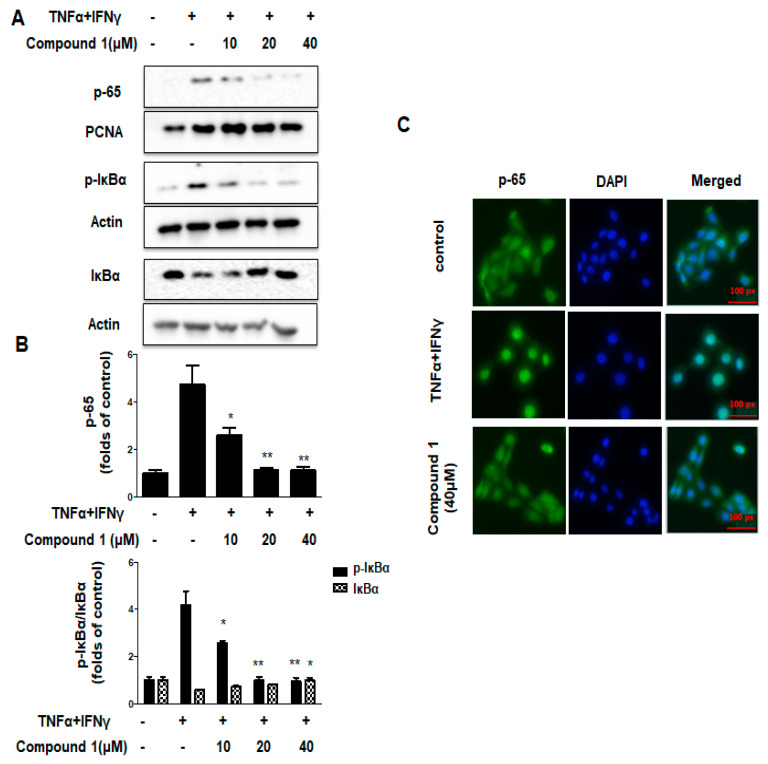
Effects of compound **1** on NF-κB signaling pathways in HaCaT cells (**A**–**C**). The expression of p65, p-IκBα, and IκBα in the fractions were determined by using Western blotting. Immunofluorescence assay was performed as described in the methods section. Data are represented as the mean ± SD (n = 3). * *p* < 0.05, ** *p* < 0.01, vs. TNF-α/IFN-γ-treated group.

In resting cells, inhibitor protein IκB forms a complex with inactive NF-κB, retaining it in the cell cytoplasm. Upon stimulation, IκB is phosphorylated and subsequently degraded by the proteasome, causes the translocation of NF-κB into the nucleus, where it regulates the transcription of specfic genes encoding proinflammatory cytokines [22,23]. In Figure 6A,B, co-treatment with TNF-α and IFN-γ significantly increased the p65 and p-IκBα levels compared with that in the control group. Pre-treatment with compound **1** decreased IκBα phosphorylation and p65 activation. The p-IκBα/IκBα ratio also decreased. Additionally, in Figure 6C, the results of immunofluorescence showed that the p65 nuclear translocation was inhibited by compound **1**, demonstrating that compound **1** can alleviate inflammation in HaCaT cells through NF-κB signaling pathways.

## 3. Discussion

Currently, systemic antihistamines, topical steroids, and immune suppressive agents are the main drugs used to treat AD. However, most patients are apprehensive about possible recurrence or side effects due to long-term treatment with these agents [23]. The search for new anti-inflammatory agents from natural sources has recently gained global interest. Fungi has been considered as special and abundant bioactive secondary metabolites [24]. In the present study, four additional compounds were isolated from the culture of fungal strain *Pleosporales* sp. SF-7343: 14-methoxyalternate C (**1**), 5-methoxy-6-methyl-biphenyl-3,4,3-triol (**2**),3,8,10-trihydroxy-4-methoxy-6-methylbenzocoumarin (**3**), and alternariol monomethyl ether (**4**). Among them, 14-methoxyalternate C showed inhibitory effects on TNF-α and IFN-γ stimulated HaCaT cells.

JAK inhibitors have been investigated as possible solutions for treating AD [24]. The inhibition of pro-inflammatory cytokine or chemokine secretion from the JAK-STAT pathway ultimately ameliorates the symptoms of AD, leading to improved quality of the patient’s life [25]. STATs are phosphorylated upon cytokine stimulation by JAK, causing the dimerization of STAT, followed by translocation of STAT to the nucleus through the nuclear membrane to combine and regulate the expression of their target genes. The imbalance in T helper (Th)-2 cells is an important immunopathological characteristics of AD. JAK-STAT pathway will activated be when the cytokine binds to their specific receptor, and then start the phosphorylation cascade on the cell cytoplasmic side, leading to the transcription of the target genes [26,27,28]. Compound **1** showed a strong regulatory effect on the activity of the JAK2/STAT3 signaling pathway and may be a good promising candidate for the treatment of AD.

Skin provides protection for our body; it is the barrier of our body and the external environments, prevent the invasion of external factors and maintain moisture [29]. Impairment of skin barrier function and the subsequently increased penetration of allergens into the skin, cause the allergic inflammatory response, this is the main feature of the Th2 inflammation in AD area [30]. FLG is a basic structural protein for the maintenance and development of the skin barrier function. It induces the outermost skin cell’s structural proteins to form tight bundles that flatten and strengthen the cells to create a strong and solid skin barrier [31]. Mutations in the FLG gene encoding filaggrin cause other atopic diseases, increased risk of ADs, and exacerbation of some conditions [32]. IVL is a protein component of human skin. It is a structural protein precursor of the keratinocyte-cornified envelope, a protective sheath of covalently crosslinked proteins that is formed during the final stages of keratinocyte differentiation [33]. In this study, compound **1** upregulated IVL expression but failed to upregulate the FLG expression, which was decreased by TNF-α and IFN-γ treatment. These results demonstrate that the protective activity of compound **1** was not exerted through modulation of filaggrin expression.

Skin inflammation is one of the key features in the pathogenesis of AD. Agents exhibiting anti-inflammatory activity could be potential therapeutic candidates for AD treatment. Our study on fungal metabolites from the Antarctic fungal strain *Pleosporales* sp. SF-7343 led to the identification of 14-methoxyalternate C (**1**), which exerted inhibitory effects on inflammation in the TNF-α and IFN-γ-induced HaCaT cells. This metabolite could be further developed as an agent to prevent inflammation in human keratinocytes.

## 4. Materials and Methods

### 4.1. Extraction and Isolation of Compound ***1*** and ***2*** from Pleosporales sp. SF-7343

The fungi culture was extracted with EtOAC, evaporated in vacuo to obtain the SF-7343V residue (3.9 g). The fungi crude extract was fractionated by a RP C_18_ flash column (4.5 × 30 cm), and a gradient elution of 20–100% (*v*/*v*) MeOH in H_2_O was set to yield 6 fractions, SF-7343V-(1–6). The fraction 4 (1.6 g) was subjected to silica gel column chromatography (3.0 × 32 cm, eluted with CH_2_Cl_2_-MeOH (100:1 to 2:1)) to yield 11 sub-fractions, SF-7343V-4-(1-11). The sub-fraction SF7343V4-5 (90 mg) was purified using a C_18_ prep HPLC [eluted with a gradient solvent system of 20–60% CH_3_CN in H_2_O (over 53 min) and 60–100% CH_3_CN in H_2_O (over 10 min)] to yield compound **1** (2.6 mg, t_R_ = 29 min). The subfraction SF7343V4-7 (42.0 mg) was separated using C_18_ prep HPLC ((36% CH_3_CN in H_2_O (over 60 min) and 60–100% CH_3_CN in H_2_O (over 10 min)) to yield compound **2** (2.6 mg, t_R_ = 18 min).

### 4.2. Extraction and Isolation of Compound **3** from Pleosporales sp. SF-7343

The fungi culture extract were evaporated to yield SF7343V(4)-RI residue (3.4 g). The fungi crude extract was fractionated by RP C_18_ flash column (5.5 × 27 cm), and a gradient elution of 20–100% (*v*/*v*) MeOH in H_2_O was set to obtain five sub-fractions SF7343V(4)-RI1 to SF7343V(4)-RI5. The fraction SF7343V(4)-RI3 (368.8 mg) was chromatographed through a silica gel column (20 × 2.7 cm) and eluted with CH_2_Cl_2_: MeOH (100:1–25:1) to yield ten sub-fractions SF7343V(4)-RI31 to SF7343V(4)-RI39. The subfraction SF7343V(4)-RI34 (33.6 mg) was purified by C18 prep HPLC (system of gradient solvent of 20–50% CH_3_CN in H_2_O for over 50 min) to obtain compound **3** (2.4 mg, t_R_ = 36 min).

### 4.3. Extraction and Isolation of Compound ***4*** from Pleosporales sp. SF-7343

The fungi culture extract were evaporated to yield the SF-7343V(567) (12.3 g). The component separation steps are the same as those mentioned in Section 4.2. Additionally, obtain five sub-fractions, SF7343V(567)-1 to SF7343V(567)-5. The sub-fraction SF7343V(567)-4 (345.8 mg) was chromatographed through a silica gel column (50 × 3 cm) and eluted with CH_2_Cl_2_:MeOH (100:0–10:1) to yield eight subfractions SF7343V(567)-41 to SF7343V(567)-48. The sub-fraction SF7343V(567)-4-3 (189.4 mg) was separated using C_18_ prep HPLC (25–65% CH_3_CN/H_2_O over 65 min) to obtain compound **4** (10.9 mg, t_R_ = 60 min).

### 4.4. Cell Culture and Reagents

HaCaT cells were cultured in DMEM media added with 10%FBS. For the source of antibody and other reagent, refer to our published articles [34].

### 4.5. MTT Assay

HaCaT cells were seeded at a density of 2 × 10^4^ cells in a 48-well plate for 24 h and then treated with compounds **1**–**4** (20–80 μM). The determination method of MTT assay is performed according to the previous paper [34].

### 4.6. Measurement of Cytokines and Chemokines

The culture supernatants were used to check the secretion of IL-6, IL-8, RANTES, and MDC by specific ELISA kits, according to the manufacturer’s instructions.

### 4.7. Extraction of Total, Nuclear, and Cytosolic Protein

HaCaT cells were pretreated with compound **1** for 3 h. For the total protein analysis, cells were lysed by RIPA buffer. For nuclear and cytoplasmic proteins, the cells were extracted by using the Nuclear Extraction Kit according to the manufacturer’s instructions. 

### 4.8. Western Blot Analysis

For the separation of protein samples using SDS-PAGE gels and transfer to NC membranes, the experimental steps are according to our published articles [34].

### 4.9. Immunofluorescence

For the translocation of NF-κB and STAT3, HaCaT cells were cultured on glass chamber slides. After treatment, the cells were fixed in paraformaldehyde, permeabilized with 0.01% TX-100, and probed first with NF-κB and STAT3 antibodies and second with FITC-labelled secondary antibodies. Then, the cells were treated with DAPI solution for 10 min and washed, and then, coverslips were covered on glass slides with an anti-fade reagent. Pictures were taken under a Nikon fluorescence microscope (ECLIPSE Ts2; Nikon Optical Co, Tokyo, Japan). 

### 4.10. Statistical Analysis

Results for each group are represented as the mean ± standard deviation (SD) (n = 3). one-way analysis of variance was carried out by the GraphPad Software. The significance results were followed by Duncan’s multiple comparison tests. Statistical significance was set to * *p* < 0.05, ** *p* < 0.01, *** *p* < 0.001 vs. the TNF-α/IFN-γ-treated groups.

## 5. Conclusions

This time, the four compounds isolated from the fungal strain *Pleosporales* sp. SF-7343, their skin anti-inflammatory effects were examined in TNF-α- and IFN-γ-treated keratinocytes. Among them, 14-methoxyalternate C showed inhibitory effects on inflammatory cytokines and chemokines, decreased ICAM-1 expression, and increased IVL expression. The anti-inflammatory effect may have been exerted through the regulation of the two signaling pathways, JAK2/STAT3 and NF-κB. These results indicated that the compounds extracted from the metabolites of the fungal strain SF-7343 has the potential to become a preventive or therapeutic agent for AD patients.

## Figures and Tables

**Table 1:** Inhibitory effects of the four isolated compounds on IL-8 and IL-6 production in TNF-α/IFN-γ-treated HaCaT cells. Results are represented as the mean ± SD (n = 3). Compounds concentration: ^a^—40 μM.

Compound	Name	Rate of Inhibition (%)	
		IL-8	IL-6
Compound **1** Compound **2** Compound **3** Compound **4**	14-methoxyalternate C 5′-methoxy-6-methyl-biphenyl-3,4,3’-triol 3,8,10-trihydroxy-4-methoxy-6-methylbenzocoumarin Altenuene	76.74 ± 5.38 ^a^ 45.90 ± 3.66 ^a^ 44.59 ± 9.58 ^a^ -	55.19 ± 4.89 ^a^ - - -

## Data Availability

The data presented in this study are available in this article. Other data supporting the findings of this study are available upon request from the corresponding author.

## Supplementary Materials

The supporting information can be downloaded at: https://www.mdpi.com/article/10.3390/ijms232314642/s1.
