# Supplementary material for: Anti-Inflammatory Effects Exerted by 14-Methoxyalternate C from Antarctic Fungal Strain Pleosporales sp. SF-7343 via the Regulation of NF-κB and JAK2/STAT3 in HaCaT Human Keratinocytes
Source: Int J Mol Sci. 2022 Nov 24;23(23):14642. doi: 10.3390/ijms232314642 (PMC9740060; doi:10.3390/ijms232314642)
Supplement: Supplementary file 1 [file ijms-23-14642-s001.zip › ijms-2013872-supplementary.pdf]

Table S1. ^{1}H and ^{13}C NMR spectroscopic data of **Compound 1** (^{1}H: 400 MHz; ^{13}C: 100 MHz).

Position	Compound 1	
	δ_H (*J* in Hz)	δ_C
1	-	171.5
2	-	108.2
3	-	162.9
4	6.20 (1H, d, *J* = 2.4)	110.8
5	-	163.6
6	6.44 (1H, d, *J* = 2.4)	100.4
7	-	144.6
8	-	134.2
9	6.53 (1H, s)	116.1
10	-	145.1
11	-	144.6
12	6.84 (1H, s)	116.3
13	-	126.9
14	3.95 (1H, d, *J* = 11.2)	72.5
	3.91 (1H, d, *J* = 11.2)	
1-OCH_3	3.39 (3H, s)	52.0
5-OCH_3	3.77 (3H, s)	55.8
14-OCH_3	3.07 (3H, s)	57.5

Table S2. ^{1}H and ^{13}C NMR spectroscopic data of **Compound 2** (^{1}H: 400 MHz; ^{13}C: 100 MHz).

Position	Compound 2	
	δ_H (*J* in Hz)	δ_C
1	-	134.4
2	6.68 (1H, s)	117.4
3	-	145.1
4	-	143.6
5	6.70 (1H, s)	118.0
6	-	129.6
1'	-	145.1
2'	6.35 (2H, d, *J* = 1.6, overlapped H-4")	109.8
3'	-	159.0
4'	6.35 (2H, d, *J* = 1.6, overlapped H-2")	100.3
5'	-	161.6
6'	6.31 (1H, d, *J* = 2.0)	107.2
6-CH_3	2.11 (3H, s)	19.8
3'-OCH_3	3.76 (3H, s)	55.5

Table S3. ^{1}H and ^{13}C NMR spectroscopic data of **Compound 3** (^{1}H: 400 MHz).

Position	Compound 3	
	δ_H (*J* in Hz)	δ_C
1	-	-
2	-	-
3	-	-
4	-	-
4a	-	-
5	6.79 (1H, s)	-
6	-	-
6a	-	-
7	7.27 (1H, d, *J* = 2.2)	-
8	-	-
9	6.54 (1H, d, *J* = 2.2)	-
10	-	-
10a	-	-
10b	-	-
6-CH_3	2.69 (3H, s)	-
4-OCH_3	3.94 (3H, s)	-

Table S4. ^{1}H and ^{13}C NMR spectroscopic data of **Compound 4** (^{1}H: 400 MHz; ^{13}C: 100 MHz).

Position	Compound 4	
	δ_H (J in Hz)	δ_C
1	-	137.8
2	-	98.4
3	-	164.1
4	6.61 (1H, d, J = 2.0)	99.1
5	-	164.7
6	7.22 (1H, d, J = 2.0)	103.3
7	-	166.2
1'	-	108.7
2'	-	152.7
3'	6.64 (1H, d, J = 2.4)	101.6
4'	-	158.8
5'	6.72 (1H, d, J = 2.4)	117.7
6'	-	138.4
6'-CH_3	2.73 (3H, s)	25.0
5-OCH_3	3.91 (3H, s)	55.8
